# The Analgesic Effect of Different Concentrations of Epidural Ropivacaine Alone or Combined With Sufentanil in Patients After Cesarean Section

**DOI:** 10.3389/fphar.2021.631897

**Published:** 2021-02-22

**Authors:** Fangfang Miao, Kunpeng Feng, Xuexin Feng, Long Fan, Yu Lang, Qingfang Duan, Ruixue Hou, Di Jin, Tianlong Wang

**Affiliations:** Department of Anesthesiology, Xuanwu Hospital, Capital Medical University, Beijing, China

**Keywords:** cesarean section, postoperative pain, analgesia, sufentanil, ropivacaine

## Abstract

**Background:** Patients experience moderate-high intensity postoperative pain after cesarean section (CS). The aim of this study was to investigate the optimal concentrations of ropivacaine and sufentanil for use in controlling pain after CS.

**Methods:** One hundred and seventy-four women undergoing elective CS were randomly allocated to four groups. Epidural analgesia was administered with 0.1% ropivacaine in the R_1_ group, 0.15% ropivacaine in the R_2_ group, a combination of 0.1% ropivacaine and 0.5 μg/ml of sufentanil in the R_1_S group, and a combination of 0.15% ropivacaine and 0.5 μg/ml of sufentanil in the R_2_S group (at a basal rate of 4 ml/h, bolus dose of 4 ml/20 min as needed). Pain scores (numerical rating scale [NRS]: 0–10 cm) at rest (NRS-R), during movement (NRS-M), and when massaging the uterus (NRS-U) were documented at 6 and 24 h. We also recorded patient satisfaction scores, time to first flatus, motor deficits, and adverse drug reactions.

**Results:** NRS (NRS-R, NRS-M, NRS-U) scores in the R_2_S group (2 [1–3], 4 [3–5], 6 [5–6], respectively) were lower than in the R_1_ group (3 [3–4], 5 [4–6], 7 [6–8], respectively) (*p* < 0.001, *p* < 0.05, *p* < 0.01, respectively) at 6 h; and patient satisfaction (9 [8–10]) was improved compared to the R_1_ group (8 [6–8]) (*p* < 0.01). The time to first flatus (18.7 ± 11.8 h) was reduced relative to the R_1_ group (25.9 ± 12.0 h) (*p* < 0.05). The time to first ambulation was not delayed (*p* > 0.05). However, the incidence of pruritus (4 [9.3%]) was increased compared to the R_2_ group (0 [0]) (*p* < 0.05) at 6 h, and the incidence of numbness (23 [53.5%], 23 [53.5%]) was increased compared to the R_1_ group (10 [23.3%], 10 [23.3%]) (all *p* < 0.01) at both 6 and 24 h.

**Conclusions:** Although we observed a higher incidence of pruritus and numbness, co-administration of 0.15% ropivacaine and 0.5 μg/ml of sufentanil administered epidurally optimized pain relief after CS, with treated subjects exhibiting lower NRS scores, shorter time to first flatus, and higher patient-satisfaction scores.

## Introduction

A surfeit of cesarean sections are performed annually worldwide. In 2016, the cesarean section (CS) rate was 31.9% (Martin et al., 2017) and 41.1% (Liang et al., 2018) in the United States and China, respectively. Despite the numerous measures used to manage pain, the incidence of inadequate analgesia after CS remains nearly 50% (Patel et al., 2017). Pain relief after CS is crucial, as poor pain relief can interfere with breast feeding and delay infant weight gain (Hirose et al., 1996). Inadequate analgesia can impair functions such as ambulation and dietary intake, and lead to complications such as thromboembolism (Leung, 2004) and postoperative ileus (inhibition of gastrointestinal motility after surgery that prevents oral intake) (Cho et al., 2017). Poor pain relief may even lead to chronic pain (Jin et al., 2016) and increased postpartum depression (Shen et al., 2020). Therefore, effective pain management after CS continues to be a relevant public health issue.

The search for the ideal method to manage postoperative pain is ongoing. Although the use of opioids is the gold standard (Kerai et al., 2017), the effects of opioids on lactation have limited intravenous opioid use—especially long-term use. Wu *et al.* found that patient-controlled epidural analgesia (PCEA) provided significantly superior analgesia compared with intravenous patient-controlled analgesia (PCIA) (Wu et al., 2005). Epidural morphine is commonly used for analgesia after CS (Carvalho and Butwick, 2017), but can produce a high incidence of pruritus, nausea, and vomiting (Youssef et al., 2014). These adverse drug reactions (ADRs) are difficult to prevent, and therefore modified regimens such as low concentrations of local anesthetics together with other opioids have been advocated to reduce opioid dosage and thereby decrease the incidence of bothersome ADRs. Epidural opioids also reduce the dosage of local anesthetics. A study by Gogarten *et al.* found that when combined with ropivacaine, sufentanil can reduce the concentration of ropivacaine required and the degree of motor blockade (Gogarten et al., 2004).

Ropivacaine has been used in labor for epidural analgesia and post-cesarean analgesia because of its lower potential for accumulation, greater sensorimotor-differential block, and increased cardiovascular safety profile (Bullingham et al., 2018; Wang et al., 2019). The breast milk/plasma concentration ratio of ropivacaine was lower than bupivacaine when administered epidurally after CS (Matsota et al., 2009), which is essential for neonatal safety. Although ropivacaine co-administered with sufentanil successfully reduced postoperative pain, few investigators have thus far evaluated the optimal concentration necessary. Therefore, we evaluated the analgesic effects by using two different concentrations (0.1% and 0.15%) of ropivacaine with or without 0.5 μg/ml of sufentanil to relieve post-cesarean pain.

## Materials and Methods

This trial was conducted at the Department of Anesthesiology, Xuanwu Hospital of Capital Medical University in China. Ethics approval (ea1/185/10) was obtained from the ethics committee of the Xuanwu Hospital of Capital Medical University. The trial was registered with the Chinese clinical trial registry (ChiCTR 1900 021740), and written informed consent was obtained on the day before surgery.

Our primary aim was to compare the analgesic efficacy of the four groups at the first 24 h after CS by a prospective, randomized, double-blinded clinical trial. Our secondary objective was to compare patient satisfaction, gastrointestinal symptoms, postsurgical recovery, and ADRs among the four groups.

### Participants

This trial included 174 women who were enrolled from March 2019 to September 2019. Women were eligible for inclusion if they were 21–45 years of age after 32 weeks of gestation (If the gestational age is less than 32 weeks, the newborn will be transferred to the children’s hospital, which may affect the mother’s mood) and at the American Society of Anesthesiology physical status classification I–II (Matsota et al., 2011), and were scheduled to undergo CS under neuraxial anesthesia. Patients with a known allergy to sufentanil and/or local anesthetics; a history of chronic pain, chronic opioid use, anxiolytic medication use, or drug and/or alcohol abuse; showing contraindications for neuraxial anesthesia; or manifested eclampsia, HELLP syndrome (hemolysis, elevated liver enzymes and low platelet count), or hemostatic disorders were excluded.

### Study Protocol

After pre-hydration with 500 ml of lactated Ringer’s solution, women underwent combined spinal-epidural anesthesia (CSEA) at the L2–L3 or L3–L4 spinal interspace in the right lateral position, receiving an injection containing between 7.5 and 10 mg of 0.5% hypobaric bupivacaine (Shanghai Harvest Pharmaceutical, Shanghai, China), depending upon their height (for a height <155 cm, 7.5 mg was administered; 155–160 cm, 8 mg; 160–165 cm, 8.5 mg; 165–170 cm, 9 mg; 170–175 cm, 9.5 mg; and >175 cm, 10 mg). After intrathecal injection, the epidural catheter was inserted up to 3 cm into the epidural space, and then patients were placed in a supine position in a left-lateral tilt. Once a bilateral sensory block to the T4 dermatome was achieved (which we tested by pinprick), the operation began. If sensory block was not achieved to the T4 level, 2% lidocaine (Shanghai Harvest Pharmaceutical, Shanghai, China) was administered into the epidural space to achieve adequate anesthesia, and the patients were excluded from further analysis.

Before skin closure, a 3-ml 2% lidocaine solution was administered into the epidural space to confirm adequate placement of the epidural catheter, and a CADD™ Administration set, Model 6,300 (Smiths Medical, St Paul, MN, United States), was then connected to each patient. Patients were randomized to the receipt of a continuous epidural infusion with PCEA of one of the four solutions using the sealed-envelope technique: a R_1_ group, 0.1% ropivacaine (AstraZeneca AB, Sodertalje, Sweden); a R_2_ group, 0.15% ropivacaine; a R_1_S group, 0.1% ropivacaine +0.5 μg/ml of sufentanil (Yichang Humanwell pharmaceutical, Hubei, China); and a R_2_S group, 0.15% ropivacaine +0.5 μg/ml of sufentanil. Dosage was a 4-ml/hour background infusion with a bolus dosage of 4 ml/20 min as needed, and a maximal dosage of 16 ml/h.

### Measurements

A blinded observer evaluated the patients. The primary outcome was pain scores as measured using a numerical rating scale (NRS) (0–10 cm: 0, no pain; 10, worst pain imaginable): NRS pain scores at rest (NRS-R), NRS pain scores during movement (NRS-M), and NRS pain scores when massaging the uterus (NRS-U) (Nie et al., 2017) at 6 and 24 h after skin closure. Overall patient satisfaction scores (0–10: 0, dissatisfied; 10, very satisfied) (Tonosu et al., 2020) were recorded 24 h. Sedation was assessed using the Ramsay Sedation Scale: 1, anxious and restless or agitated or both; 2, cooperative, tranquil, and oriented; 3, somnolent, responds to commands only; 4, asleep, shows brisk response to loud auditory stimulus or light glabellar tap; 5, asleep, shows sluggish response to loud auditory stimulus or light glabellar tap; and 6, asleep, shows no response to loud auditory stimulus or light glabellar tap (Nie et al., 2017). The time of first ambulation (able to walk with help) and the time of first flatus were recorded. If the patient persisted with pain, flurbiprofen axetil (Beijing Tide pharmaceutical, Beijing, China) was intravenously injected and documented. We surveilled for ADRs including pruritus, nausea and vomiting, numbness, headache, and dizziness. We also recorded any hypotension (20% below baseline blood pressure, baseline blood pressure was defined as the blood pressure in the preoperative assessment or the usual blood pressure at home) (Siddik-Sayyid et al., 2014; Nie et al., 2017) or respiratory depression (respiratory rate <8 breaths/min).

### Statistical Analysis

In this study, analgesic efficacy was the primary outcome. A power analysis showed that 42 patients in each group would provide 80% of the power needed to detect differences among the four groups.

We performed all statistical analyses with SPSS 25.0 software, and statistical significance was set at *p* = 0.05 (2-sided). A Shapiro-Wilk test, Q-Q plot, and p-p plot were used to confirm normality for continuous variables. All normally distributed continuous variables are reported as means ± SD, and non-normally distributed continuous variables are described as medians (25–75% interquartile range [IQR]). One-way ANOVA or Kruskal-Wallis methods were used to compare the differences between/among groups, and Tukey’s and Dscf tests were used to perform the post-hoc analyses. The Chi-squared test was used to compare the differences between categorical variables, and data are expressed as N (%) patients.

## Results

**FIGURE 1:**
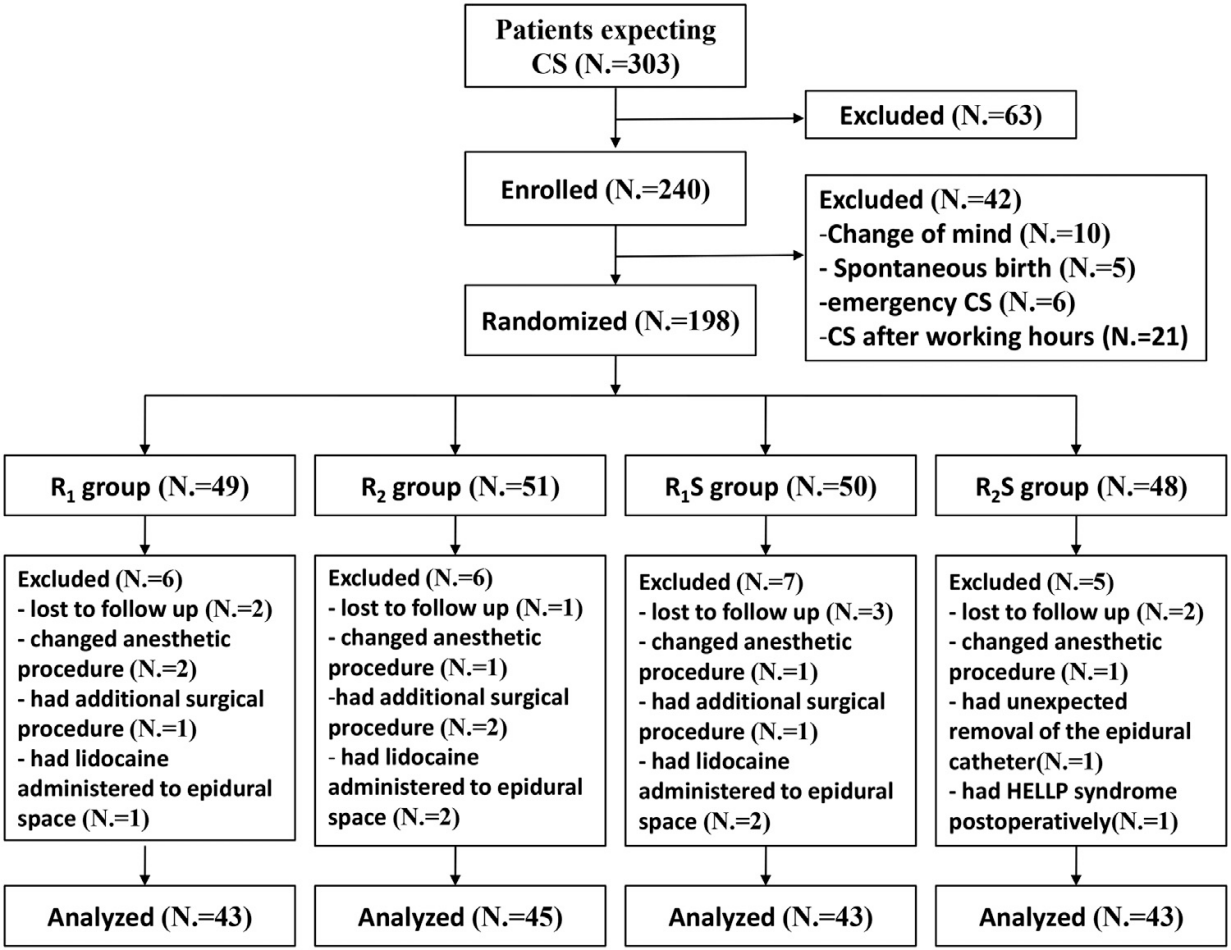
Study flow-chart. R_1_ group, 0.1% ropivacaine; R_2_ group, 0.15% ropivacaine; R_1_S group, 0.1% ropivacaine +0.5 μg/ml of sufentanil; R_2_S group, 0.15% ropivacaine +0.5 μg/ml of sufentanil. CS, cesarean section.

Of the 303 patients screened for eligibility, we enrolled 240 patients, and 42 patients were excluded prior to randomization (Figure 1). One hundred and ninety-eight patients were subsequently randomly allocated to four groups. After randomization, 24 patients were excluded (Figure 1). We ultimately analyzed 43 patients in the R_1_ group, 45 in the R_2_ group, 43 in the R_1_S group, and 43 in the R_2_S group (Figure 1).

### Patient Demographics and Surgical Characteristics

Baseline characteristics for the four groups are shown in Table 1. There were no statistically significant differences among the four groups with respect to age, height, weight, gestational age at delivery, parity, the duration of surgery, infusion volume, estimated blood loss, the puncture interspace location, or the dose of intrathecal 0.5% hypobaric bupivacaine (all *p* > 0.05).

**TABLE 1:** Patient characteristics and surgical data.

	R_1_ (43)	R_2_ (45)	R_1_S (43)	R_2_S (43)	*p* values
Age (years)	34.1 ± 4.2	34.1 ± 3.7	34.4 ± 4.1	35.3 ± 4.6	0.445
Height (cm)	162.9 ± 4.9	161.6 ± 4.9	161.3 ± 6.0	162.0 ± 6.0	0.536
Weight (kg)	79.0 ± 12.9	74.3 ± 10.4	76.3 ± 13.9	76.8 ± 10.5	0.341
Gestational age at delivery (weeks)	38 (37–39)	38 (38–39)	38 (38–38)	38 (38–39)	0.191
Parity (number)					0.645
1	26 (26.0%)	29 (29.0%)	24 (24.0%)	21 (21.0%)	
2	17 (24.6%)	15 (21.7%)	17 (24.6%)	20 (29.1%)	
3	0 (0.00%)	1 (20.0%)	2 (40.0%)	2 (40.0%)	
Duration of surgery (min)	44 (38–52)	39 (32–43)	41 (36–55)	40 (34–49)	0.071
I V infusion volume (ml)	800 (800–900)	800 (700–800)	800 (700–1,100)	800 (700–900)	0.543
Estimated blood loss (ml)	300 (200–300)	200 (200–300)	200 (200–400)	200 (200–300)	0.859
Puncture interspace location					0.685
L3–4	6 (21.4%)	9 (32.1%)	5 (17.9%)	8 (28.6%)	
L2–3	37 (25.3%)	36 (24.7%)	38 (26.0%)	35 (24.0%)	
Dose of intrathecal 0.5% bupivacaine (mg)	1.5 (1.5–1.6)	1.5 (1.5–1.6)	1.5 (1.5–1.6)	1.5 (1.5–1.6)	0.953

R_1_ group, 0.1% ropivacaine; R_2_ group, 0.15% ropivacaine; R_1_S group, 0.1% ropivacaine +0.5 μg/ml of sufentanil; R_2_S group, 0.15% ropivacaine +0.5 μg/ml of sufentanil. Data are expressed as means ± SD, medians (25–75% IQR) or N (%) patients.

### Postoperative NRS Scores

**FIGURE 2:**
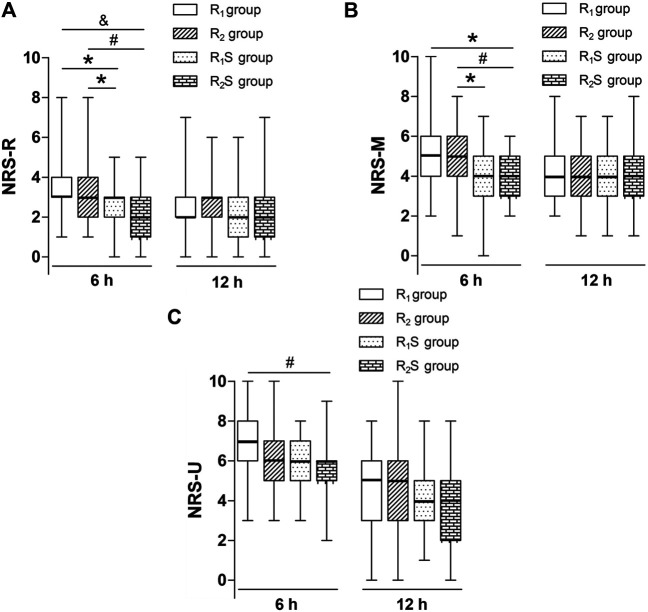
**(A)** Numerical rating scale of pain scores at rest (NRS-R) at 6 and 24 h after skin closure. **(B)** Numerical rating scale of pain scores during movement (NRS-M) at 6 and 24 h after skin closure. **(C)** Numerical rating scale of pain scores when massaging the uterus (NRS-U) at 6 h and 24 h after skin closure. R_1_ group, 0.1% ropivacaine; R_2_ group, 0.15% ropivacaine; R_1_S group, 0.1% ropivacaine +0.5 μg/ml of sufentanil; R_2_S group, 0.15% ropivacaine +0.5 μg/ml of sufentanil. Data are expressed as medians (25–75% IQR). *p < 0.05, ^#^ *p* < 0.01, ^&^ *p* < 0.001.

Six hours after skin closure, NRS-R scores in the R_1_S (3 [2–3]) and R_2_S (2 [1–3]) groups were lower than those in either the R_1_ (3 [3–4]) (*p* < 0.05, *p* < 0.001, respectively) or R_2_ groups (3 [2–4]) (*p* < 0.05, *p* < 0.01, respectively). At 24 h after skin closure, there were no statistically significant differences in NRS-R scores among the four groups (*p* > 0.05) (Figure 2A).

We noted that at 6 h after skin closure, NRS-M scores in the R_1_S group (4 [3–5]) were lower than those in the R_2_ group (5 [4–6]) (*p* < 0.05), and NRS-M scores in the R_2_S group (4 [3–5]) were lower than in the R_1_ (5 [4–6]) (*p* < 0.05) and R_2_ (5 [4–6]) (*p* < 0.01) groups. By 24 h after CS, there were no longer any statistically significant differences in NRS-M scores among the four groups (*p* > 0.05) (Figure 2B).

By 6 h after skin closure, NRS-U scores in the R_2_S group (6 [5–6]) were lower than those in the R_1_ group (7 [6–8]) (*p* < 0.01), but there was no statistically significant differences between R_1_S and R_1_ groups (*p* > 0.05). By 24 h after CS, there were no statistically significant differences in scores among the four groups (*p* > 0.05) (Figure 2C).

### Patient Satisfaction

**FIGURE 3:**
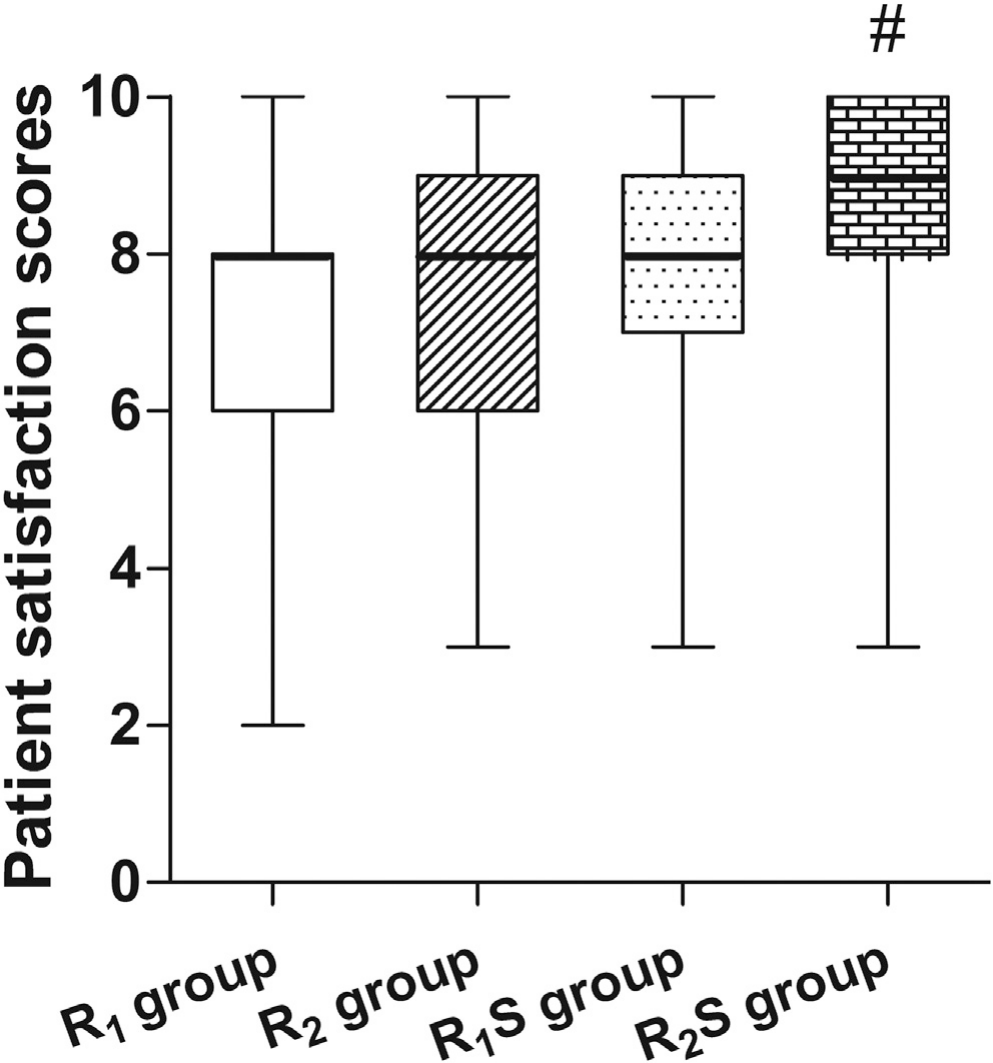
Patient’s overall satisfaction measured 24 h after cesarean section. R_1_ group, 0.1% ropivacaine; R_2_ group, 0.15% ropivacaine; R_1_S group, 0.1% ropivacaine +0.5 μg/ml of sufentanil; R_2_S group, 0.15% ropivacaine +0.5 μg/ml of sufentanil. Data are expressed as medians (25–75% IQR). ^#^ *p* < 0.01.

Patient satisfaction scores were significantly higher in the R_2_S group (9 [8–10]) relative to the R_1_ group (8 [6–8]) (*p* < 0.01). There were no statistically significant differences between any of other two groups (all *p* > 0.05) (Figure 3).

### Gastrointestinal Function

**FIGURE 4:**
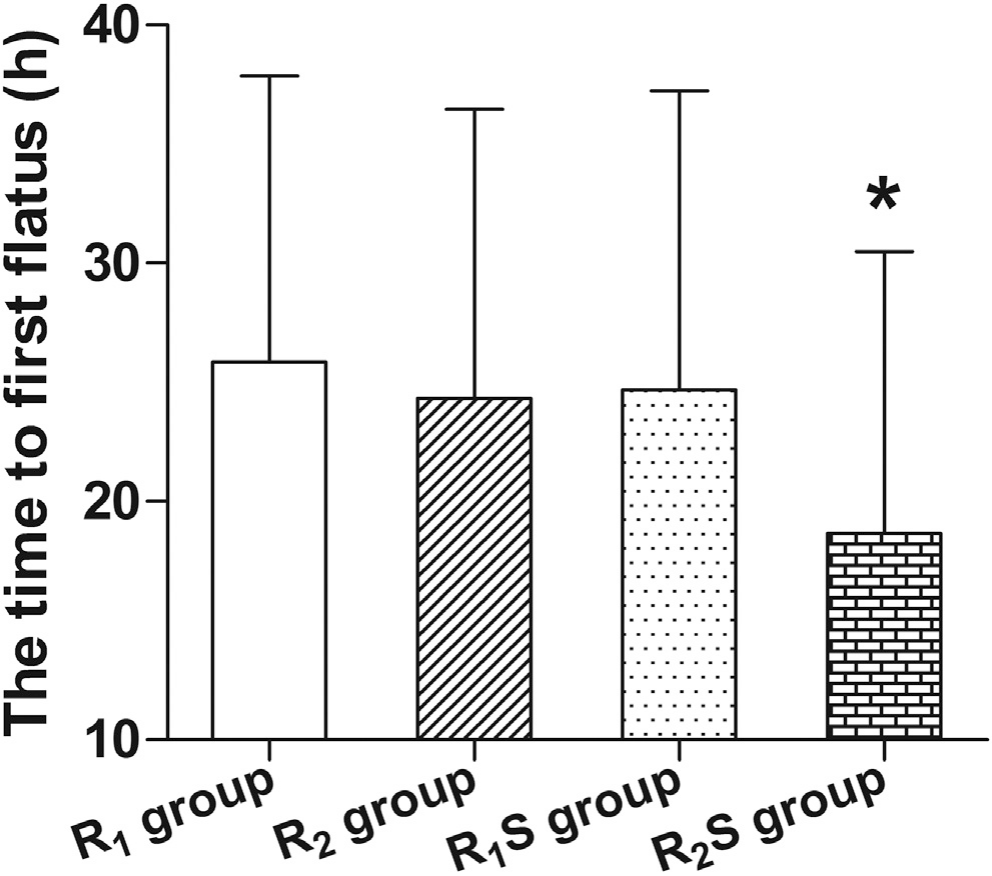
Time to patient’s first flatus after cesarean section. R_1_ group, 0.1% ropivacaine; R_2_ group, 0.15% ropivacaine; R_1_S group, 0.1% ropivacaine +0.5 μg/ml of sufentanil; R_2_S group, 0.15% ropivacaine +0.5 μg/ml of sufentanil. Data are expressed as means ± SD. *p < 0.05.

The time to patient’s first flatus was shorter in the R_2_S group (18.7 ± 11.8 h) compared to the R_1_ group (25.9 ± 12.0 h) (*p* < 0.05) (Figure 4).

### Requirements for Concomitant Analgesics

The number of self-administered doses of the patient-controlled analgesia (PCA) in the R_2_S group (0 [0–2]) was less than in the R_1_ (1 [0–3]) (*p* < 0.05) and R_2_ groups (1 [0–4]) (*p* < 0.05), and that was less in the R_1_S group (0 [0–2]) than in the R_2_ group (1 [0–4]) (*p* < 0.05) at 6 h, while there were no statistically significant differences among the groups at 24 h (*p* > 0.05). There were no statistically significant differences among the groups regarding the time when highest pain scores were recorded (*p* > 0.05) or the number of patients who requested supplementary analgesia, either at 6 or 24 h (all *p* > 0.05) (Table 2).

**TABLE 2:** Maternal follow-up.

	R_1_ (43)	R_2_ (45)	R_1_S (43)	R_2_S (43)	*p* values
Time until greatest pain score experienced (h)	9.8 ± 5.9	9.1 ± 5.6	11.0 ± 6.8	10.2 ± 7.4	0.618
Number of self-administered doses					
6 h	1 (0–3)	1 (0–4)	0 (0–2)*^3^	0 (0–2) *^1,2^	0.026
24 h	0 (0–2)	0 (0–1)	0 (0–0)	0 (0–1)	0.199
Number of patients who requested supplementary analgesia					
6 h	3 (7.0%)	2 (4.4%)	4 (9.3%)	0 (0)	0.243
24 h	0 (0)	0 (0)	1 (2.3%)	0 (0)	0.382
Time to first ambulation (h)	25.3 (23.7–28.0)	26.7 (24.6–28.1)	27.3 (25.0–28.5)	24.8 (22.5–27.5) *^4^	0.019
Ramsay sedation scale scores					
6 h					0.371
1	3 (7.0%)	2 (4.4%)	0 (0)	1 (2.3%)	
2	40 (93.0%)	43 (95.6%)	43 (100%)	41 (95.4%)	
3	0 (0)	0 (0)	0 (0)	1 (2.3%)	
4	0 (0)	0 (0)	0 (0)	0 (0)	
5	0 (0)	0 (0)	0 (0)	0 (0)	
6	0 (0)	0 (0)	0 (0)	0 (0)	
24 h					0.886
1	3 (7.0%)	2 (4.4%)	0 (0)	1 (2.3%)	
2	40 (93.0%)	42 (93.4%)	40 (93.0%)	41 (95.3%)	
3	0 (0)	1 (2.2%)	1 (2.3%)	0 (0)	
4	0 (0)	0 (0)	0 (0)	0 (0)	
5	0 (0)	0 (0)	0 (0)	0 (0)	
6	0 (0)	0 (0)	0 (0)	0 (0)	

R_1_ group, 0.1% ropivacaine; R_2_ group, 0.15% ropivacaine; R_1_S group, 0.1% ropivacaine +0.5 μg/ml of sufentanil; R_2_S group, 0.15% ropivacaine +0.5 μg/ml of sufentanil. Data are expressed as means ± SD, medians (25–75% IQR) or N (%) patients. *^1^p < 0.05: R_2_S group vs. R_1_ group, *^2^p < 0.05: R_2_S group vs. R_2_ group, *^3^p < 0.05: R_1_S group vs. R_2_ group, *^4^p < 0.05: R_1_S group vs. R_2_S group.

### Recovery of Motor Function

The time to patient’s first ambulation was shorter in the R_2_S group (24.8 [22.5–27.5 h]) than in the R_1_S group (27.3 [25.0–28.5 h]) (*p* < 0.05). There were no statistically significant differences between any of other two groups (all *p* > 0.05) (Table 2).

### Sedation Scores

We observed no statistically significant differences in Ramsay Sedation Scale scores among the four groups (*p* > 0.05) (Table 2).

### Adverse Drug Reactions

Patients in the R_1_S group (4 [9.3%]) had a higher incidence of pruritus at 6 h compared to the R_1_ (0 [0]) (*p* < 0.05) and R_2_ groups (0 [0]) (*p* < 0.05), while patients in the R_2_S group (4 [9.3%]) exhibited a higher incidence of pruritus at 6 h compared to the R_2_ group (0 [0]) (*p* < 0.05). The incidence of numbness in the R_2_ (22 [48.9%], *p* < 0.05; 22 [48.9%], *p* < 0.05) and R_2_S groups (23 [53.5%], *p* < 0.01; 23 [53.5%], *p* < 0.01) was greater than in the R_1_ group (10 [23.3%], 10 [23.3%]) at both 6 and 24 h. There were no statistically significant differences among the groups regarding other ADRs, including nausea and vomiting, headache, dizziness, hypotension, or respiratory depression (all *p* > 0.05) (Table 3).

**TABLE 3:** Adverse effects.

	R_1_ (43)	R_2_ (45)	R_1_S (43)	R_2_S (43)	*p* values
Pruritus					
6 h	0 (0)	0 (0)	4 (9.3%)*^3,4^	4 (9.3%)*^2^	0.035
24 h	0 (0)	0 (0)	3 (7.0%)	4 (7.0%)	0.095
Numbness					
6 h	10 (23.3%)	22 (48.9%)*^5^	17 (39.5%)	23 (53.5%)*^1^	0.023
24 h	10 (23.3%)	22 (48.9%)*^5^	17 (39.5%)	23 (53.5%)*^1^	0.023
Nausea and vomiting					
6 h	1 (2.3%)	1 (2.2%)	2 (4.7%)	1 (2.3%)	0.885
24 h	0 (0)	1 (2.2%)	0 (0)	1 (2.3%)	0.577
Headache					
6 h	0 (0)	1 (2.2%)	0 (0)	0 (0)	0.410
24 h	1 (2.3%)	0 (0)	1 (2.3%)	0 (0)	0.558
Dizziness					
6 h	0 (0)	1 (2.2%)	1 (2.3%)	1 (2.3%)	0.800
24 h	0 (0)	2 (4.4%)	0 (0)	0 (0)	0.122
Hypotension					
6 h	0 (0)	0 (0)	0 (0)	1 (2.3%)	0.382
24 h	0 (0)	0 (0)	0 (0)	0 (0)	1.000
Respiratory depression					
6 h	0 (0)	0 (0)	0 (0)	0 (0)	1.000
24 h	0 (0)	0 (0)	0 (0)	0 (0)	1.000

R_1_ group, 0.1% ropivacaine; R_2_ group, 0.15% ropivacaine; R_1_S group, 0.1% ropivacaine +0.5 μg/ml of sufentanil; R_2_S group, 0.15% ropivacaine +0.5 μg/ml of sufentanil. Data are expressed as N (%) patients. *^1^p < 0.01: R_2_S group vs. R_1_ group; *^2^p < 0.05: R_2_S group vs. R_2_ group; *^3^p < 0.05: R_1_S group vs. R_1_ group; *^4^p < 0.05: R_1_S group vs. R_2_ group; *^5^p < 0.05: R_2_ group vs. R_1_ group.

## Discussion

Postoperative analgesia after CS is challenging since we need not only to consider maternal comfort, but the anesthetic technique should also have no ADRs on the newborn. Epidural morphine is commonly used for analgesia after CS (Carvalho and Butwick, 2017), but there is a concomitantly higher incidence of pruritus, nausea and vomiting (Youssef et al., 2014). As these ADRs diminish overall patient satisfaction, we advocate the use of low concentrations of local anesthetics with other opioids so as to reduce opioid dosage and thereby decrease the incidence of ADRs.

The present study indicated that co-administration 0.15% ropivacaine with 0.5 μg/ml of sufentanil epidurally alleviated CS pain at rest, during movement, and when massaging the uterus, and it reduced the number of self-administered doses of the PCA compared to 0.1% ropivacaine or 0.15% ropivacaine at 6 h. Moreover, it improved overall patient satisfaction scores and reduced the time to first flatus compared to 0.1% ropivacaine. The time to first ambulation was not delayed. However, co-administered with sufentanil increased the incidence of pruritus compared to ropivacaine only at 6 h. Higher concentration of ropivacaine increased the incidence of numbness compared to lower concentration at both 6 and 24 h, whether or not it was co-administered with sufentanil. We believe that although a higher incidence of pruritus and numbness was demonstrated, co-administration of 0.15% ropivacaine with 0.5 μg/ml of sufentanil epidurally may be the better choice for pain relief relative to the other groups due to the lower NRS-R, NRS-M, and NRS-U scores, shorter time to first flatus, and higher patient-satisfaction scores.

Published guidelines advocate for fentanyl and sufentanil for epidural analgesia (Plante and Gaiser, 2017). The authors of a systematic review found that compared with fentanyl, sufentanil was more effective in extending the duration of analgesia when used in CSEA during labor (Zhi et al., 2020). Ropivacaine blocks conduction in the sensory and motor nerves, while sufentanil interrupts pain transmission in the dorsal horn. Therefore, adding sufentanil to ropivacaine may synergistically prolong the duration of sensory block (Parpaglioni et al., 2009). In the present study, ropivacaine co-administered with sufentanil relieved the pain not only at rest but also during movement compared to ropivacaine only at 6 h. However, when massage the uterus, 0.15% ropivacaine with sufentanil showed an improved analgesic effect, while 0.1% ropivacaine with sufentanil did not show improvement compared with 0.1% ropivacaine only at 6 h. All these results indicated that a higher concentration of ropivacaine (0.15%) with sufentanil produced a stronger analgesic effect.

Although NRS scores were significantly lower in the R_2_S group relative to the other groups, the analgesic effect was not satisfactory in any group when massage the uterus. Carvalho et al. found that patients tolerated moderate pain rather than exposing their baby to any possible harmful effects of analgesics (Carvalho et al., 2005), and others found that pain was not the only factor that determined overall patient satisfaction (Schyns-Van Den Berg et al., 2015); these may be reasons for the high overall satisfaction scores we received. In our study, the rare use of supplementary analgesia may be also attributed—at least partially—to the patients’ fear of exposing their baby to any possible harmful analgesics (Carvalho et al., 2005).

Postoperative pain increases the secretion of catecholamines, and sympathetic stimulation prevents the release of acetylcholine (Cho et al., 2017), thus inhibiting the recovery of gastrointestinal function; this may be the reason that time to first flatus in the R_1_, R_2_, and R_1_S groups was longer than that in the R_2_S group.

Early movement hastens postpartum recovery (Macones et al., 2019). Epidural analgesia with local anesthetics may delay ambulation of the mother, but when administered at low concentrations, the probability is small (Chen et al., 2014). In our study, the time to first ambulation in the R_2_S group was shorter than R_1_S group, this maybe attribute to the better analgesic effect in the R_2_S group than in the R_1_S group. The time to first ambulation in the R_2_S group was not delayed compared with the other groups. We attribute this to better analgesic effect and the administration of low-concentration ropivacaine (0.1% or 0.15%). Other investigators also reported similar results, Liu *et al.* found that 0.2% ropivacaine induced a 30% incidence in motor block, but not in those individuals who received 0.05% or 0.1% ropivacaine (Liu et al., 1999).

Complications of neuraxial drugs are the results of drug absorption into the blood circulation or presence in cerebrospinal fluid, or both. The principal ADRs observed in the present study were numbness and pruritus. The incidence of numbness was higher in the R_2_ and R_2_S groups when compared with the R_1_ group at both 6 h and 24 h, indicating that higher concentration of ropivacaine (0.15%) increased numbness.

The incidence of pruritus was higher in the R_2_S group relative to the R_2_ group, and that was higher in the R_1_S group compared to the R_1_ and R_2_ groups at 6 h. But the incidence of pruritus in the present study was overall low (9.3% and 7.0% at 6 and 24 h, respectively, in the R_2_S group; and 9.3% and 7.0% at 6 and 24 h, respectively, in the R_1_S group). By contrast, the incidence of epidural morphine (2.5 mg)-induced pruritus was 44% (Youssef et al., 2014). Our use of a small effective dose of sufentanil and the addition of ropivacaine might explain the low incidence of pruritus. Cai *et al.* reported that when ropivacaine was combined with sufentanil for labor analgesia, the incidence of pruritus was 3.3% (lower than what we reported herein), which may be due to the lower concentration of sufentanil (0.3 μg/ml) the authors used (Cai et al., 2020). Pruritus may, at times, be more unpleasant for patients than pain, from this perspective, epidural sufentanil appears to be better than morphine.

With respect to other ADRs such as nausea and vomiting, headache, dizziness, hypotension, and respiratory depression, there were no significant differences among the four groups.

There were some limitations to our study. First, we did not assess the possible preoperative confounding factors for pain after CS (Pan et al., 2013). However, the previous pain experience due to a preceding CS was evenly distributed among the four groups. Second, the observation period was the initial 24 h, and we recommend that future studies lengthen the observation time to 48 h or even longer. Third, in the present study, the incidence of urinary retention was not recorded, as the urinary catheter was inserted prior to surgery and removed after patients could walk with assistance in the ward.

## Conclusions

Although the ADRs (pruritus and numbness) were greater in the R_2_S group, by significantly reducing the pain scores and the time to first flatus, patients reported higher satisfaction scores in the R_2_S group relative to other groups. In conclusion, the epidural co-administration of 0.15% ropivacaine and 0.5 μg/ml of sufentanil (at a 4 ml/h background infusion with a bolus dosage of 4 ml/20 min as needed) may be the better choice for post-cesarean analgesia compared to the other three regimens investigated in this study.

## Data Availability

The raw data supporting the conclusions of this article will be made available by the authors, without undue reservation.
